# Robustness of Mammalian Gut Microbiota to Humanization in Captivity

**DOI:** 10.3389/fevo.2021.785089

**Published:** 2022-01-03

**Authors:** Brian K. Trevelline, Andrew H. Moeller

**Affiliations:** 1 Cornell Lab of Ornithology, Cornell University, Ithaca, NY, United States,; 2 Department of Ecology and Evolutionary Biology, Cornell University, Ithaca, NY, United States

**Keywords:** mammals, conservation, metagenomics, microbiome, bacteria, transmission

## Abstract

In mammals, the composition of the gut microbiota is associated with host phylogenetic history, and host-lineage specific microbiota have been shown, in some cases, to contribute to fitness-related traits of their hosts. However, in primates, captivity can disrupt the native microbiota through a process of humanization in which captive hosts acquire gut microbiota constituents found in humans. Despite the potential importance of this process for the health of captive hosts, the degree to which captivity humanizes the gut microbiota of other mammalian taxa has not been explored. Here, we analyzed hundreds of published gut microbiota profiles generated from wild and captive hosts spanning seven mammalian families to investigate the extent of humanization of the gut microbiota in captivity across the mammalian phylogeny. Comparisons of these hosts revealed compositional convergence between captive mammal and human gut microbiota in the majority of mammalian families examined. This convergence was driven by a diversity of microbial lineages, including members of the Archaea, *Clostridium*, and *Bacteroides*. However, the gut microbiota of two families—Giraffidae and Bovidae—were remarkably robust to humanization in captivity, showing no evidence of gut microbiota acquisition from humans relative to their wild confamiliars. These results demonstrate that humanization of the gut microbiota is widespread in captive mammals, but that certain mammalian lineages are resistant to colonization by human-associated gut bacteria.

## INTRODUCTION

The gut microbial communities of wild mammals tend to reflect their hosts’ phylogenetic histories. Across a diversity of mammalian taxa, gut microbial communities are strongly associated with the evolutionary histories of their hosts, in that compositional divergence of the gut microbiota increases with host phylogenetic distance (termed “phylosymbiosis”) (Ley et al., 2008; Ochman et al., 2010; Muegge et al., 2011; Brooks et al., 2016; Moeller et al., 2017; Lim and Bordenstein, 2020). Moreover, there is mounting evidence that certain host-associated microbiota have co-diversified with their hosts (Moeller et al., 2016; Groussin et al., 2017, 2020), indicating the maintenance of specific relationships between gut bacterial and host lineages over evolutionary time. These phylogenetic patterns in the gut microbiota suggest the possibility co-evolution and co-adaptation between gut bacteria and mammals, and experimental evidence in rodents has provided some evidence for this hypothesis. For example, germ-free house mice inoculated with non-native gut microbiota from other species of rodent display stunted immune maturation and growth rates relative to germ-free mice inoculated with a native house-mouse microbiota (Chung et al., 2012; Moeller et al., 2019). These studies suggest that disruption of ancient host-microbe associations can result in adverse fitness consequences for the host.

In contrast to the patterns observed in wild mammals, captive mammals can lose their species-specific gut microbiota and acquire certain gut microbiota constituents found in humans. For example, the gut microbiota of captive primates are more compositionally similar to humans than are those of wild conspecifics (Clayton et al., 2016; Houtz et al., 2021). Humanization of the gut microbiota in captivity may lead to a mismatch between host physiologies and their microbial environments, potentially contributing to the health issues observed in captive mammals such as pathogenic bacterial infections (Wasimuddin et al., 2017) and gastrointestinal dysfunction (Terio et al., 2005). Importantly, attempts to eliminate potentially pathogenic bacteria with antibiotics may further exacerbate adverse health outcomes, as these compounds also reduce the abundance of beneficial microbiota (Dahlhausen et al., 2018). Therefore, humanization of the gut microbiota presents a potential threat to the health of captive mammals and may subvert conservation efforts involving captive breeding and/or reintroductions into the wild (Trevelline et al., 2019). Nevertheless, to date the humanization of gut microbiota has only been investigated in captive primates, and the degree to which captivity humanizes the gut microbiota of other mammalian taxa has not been investigated.

Here, we leverage hundreds of published gut microbiota profiles generated from wild and captive mammals (representing eight families), as well as industrialized and non-industrialized human populations to test the hypotheses: (1) that captivity humanizes the mammalian gut microbiota, and (2) that the degree of humanization varies across mammalian families. Our analyses provided evidence of gut microbiota humanization in the majority of mammalian families examined. However, the gut microbiota of two mammalian families—Giraffidae and Bovidae—were robust to humanization in captivity, showing no evidence of gut microbiota acquisition from humans relative to their wild confamiliars. These results demonstrate that susceptibility of the gut microbiota to humanization appears to be widespread in mammals, but that certain mammalian lineages are resistant to colonization by human-associated gut bacteria.

## MATERIALS AND METHODS

### Processing and Merging 16S rDNA Sequence Data

To assess the evidence for gut microbiota humanization in captive mammals, we merged publicly available V4 16S rRNA gene datasets from wild and captive mammals (McKenzie et al., 2017) and humans (Yatsunenko et al., 2012). These datasets were generated from bead-beating DNA extraction protocol implemented by the same research group, thereby minimizing potential study effects that could confound downstream meta-analyses. Raw sequences were merged into a single analysis using the University of California, San Diego qiita webserver (Gonzalez et al., 2018), and processed for quality using “split libraries” with the following parameters: sequence_max_*n* = 0, min_per_read_length_fraction = 0.75, max_bad_run_length = 3, and phred_quality_threshold = 3. Filtered sequences were trimmed to a common length of 100 bp to enable comparisons across datasets. Filtered and trimmed sequences were then collapsed into Amplicon Sequence Variants (ASVs) using deblur (Amir et al., 2017) as implemented in qiita. ASVs were determined with Deblur using following parameters: Mean per nucleotide error rate = 0.005, Indel probability = 0.01, Minimum dataset-wide read threshold = 0, Minimum per-sample read threshold = 2, Maximum number of indels = 3. ASVs were classified to taxonomic ranks using the Silva 138 reference (Quast et al., 2012). All samples were rarefied to a common depth of 10,000 reads for downstream analyses.

### Beta Diversity Analyses

We calculated the beta diversity dissimilarities between all pairs of samples to test the hypothesis that the gut microbiota of captive mammals was more compositionally similar to humans than was the gut microbiota of wild mammals from the same family. To focus our analyses on differences in community memberships among samples, we calculated Sorensen-Dice distances in QIIME2 (Bolyen et al., 2019)—which in this context measures microbiota dissimilarity between pairs of samples based on the presence or absence of ASVs. Principal coordinates were calculated from the beta diversity dissimilarity matrix using “qiime diversity pcoa,” and samples were plotted against the first two PCs.

### Associations Between Beta Diversity and Host Phylogeny in Wild and Captive Mammals

Using the rarefied ASV table described above, we collapsed microbial inventories from wild and captive hosts in the same family to test the hypothesis that captivity altered the association between gut microbiota composition and host phylogenetic history (i.e., phylosymbiosis). This dataset also included average ASV relative abundances of *Homo sapiens* from Malawi, Venezuela, and the United States. ASVs were averaged across wild or captive hosts in the same family using the command “feature-table group” with “p-mode” = “mean-ceiling” in QIIME2. Average relative abundance of ASVs were used to calculate beta diversity dissimilarity using the binary Sorensen-Dice metric in QIIME2. We used Sorensen-Dice dissimilarities to produce microbial dendrograms using UPGMA hierarchal clustering in QIIME1 (Caporaso et al., 2010). We used the program TreeCmp (Bogdanowicz et al., 2012) and a previously published Python script (Brooks et al., 2016) to investigate the impact of humanization on patterns of phylosymbiosis among captive mammals. We tested for patterns of phylosymbiosis by comparing gut microbiota dendrograms for wild and captive mammals with mammalian host phylogeny (downloaded from TimeTree; Kumar et al., 2017) *via* the rooted Robinson–Foulds metric with 10,000 random trees. This approach produces a normalized score between 0 (complete congruence) and 1 (complete incongruence). *P*-values were determined by the probability of 10,000 randomized dendrogram topologies yielding equivalent or more congruent phylosymbiotic patterns than the actual microbiota dendrogram.

### Assessing Humanization of Captive Gut Microbiota Within Mammalian Families

To test for significant differences in dissimilarity between wild mammal microbiota vs. human microbiota and captive mammal microbiota vs. human microbiota, we employed non-parametric Monte-Carlo permutation tests using “make_distance_boxplots.py” as implemented in QIIME1.9. These analyses tested whether the microbiota of captive mammals displayed reduced compositional similarity (based on binary Sorensen-Dice dissimilarities) to microbiota of humans relative to the microbiota of wild-living mammals from the same taxonomic family. Non-parametric *p*-values from these tests were calculated based on 999 permutations.

### Phylogenetic Visualizations

Phylogenetic trees of ASVs were constructed using SEPP insertion against the Greengenes 13–8 reference phylogeny (DeSantis et al., 2006) as implemented in QIIME2. Trees were subsequently pruned in R using the package “phytools” (Revell, 2012) to remove reference sequences. Trees and distributions of ASVs were plotted with EMPress (Cantrell et al., 2021) as implemented in QIIME2.

## RESULTS

### 16S rDNA Datasets

The combined dataset contained gut microbiota profiles from 657 fecal samples collected from humans and other mammals, including 45,394 Amplicon Sequence Variants (ASVs) represented by 569,790,829 reads. In addition to humans (*n* = 528; Family Hominidae), mammalian families represented in our merged dataset included Bovidae (wild, captive [total] *n* = 11, 19 [30]), Canidae (*n* = 4, 5 [9]), Cercopithecidae (*n* = 33, 8 [41]), Equidae (*n* = 9, 22 [31]), Giraffidae (*n* = 2, 4 [6]), Orycteropodidae (*n* = 5, 18 [23]), and Rhinocerotidae (*n* = 4, 9 [13]). A complete list of samples and corresponding metadata is presented in Supplementary Table 1. The relative abundances of ASVs across samples are presented in Supplementary Table 2.

### Effects of Captivity on Patterns of Phylosymbiosis

**FIGURE 1 |:**
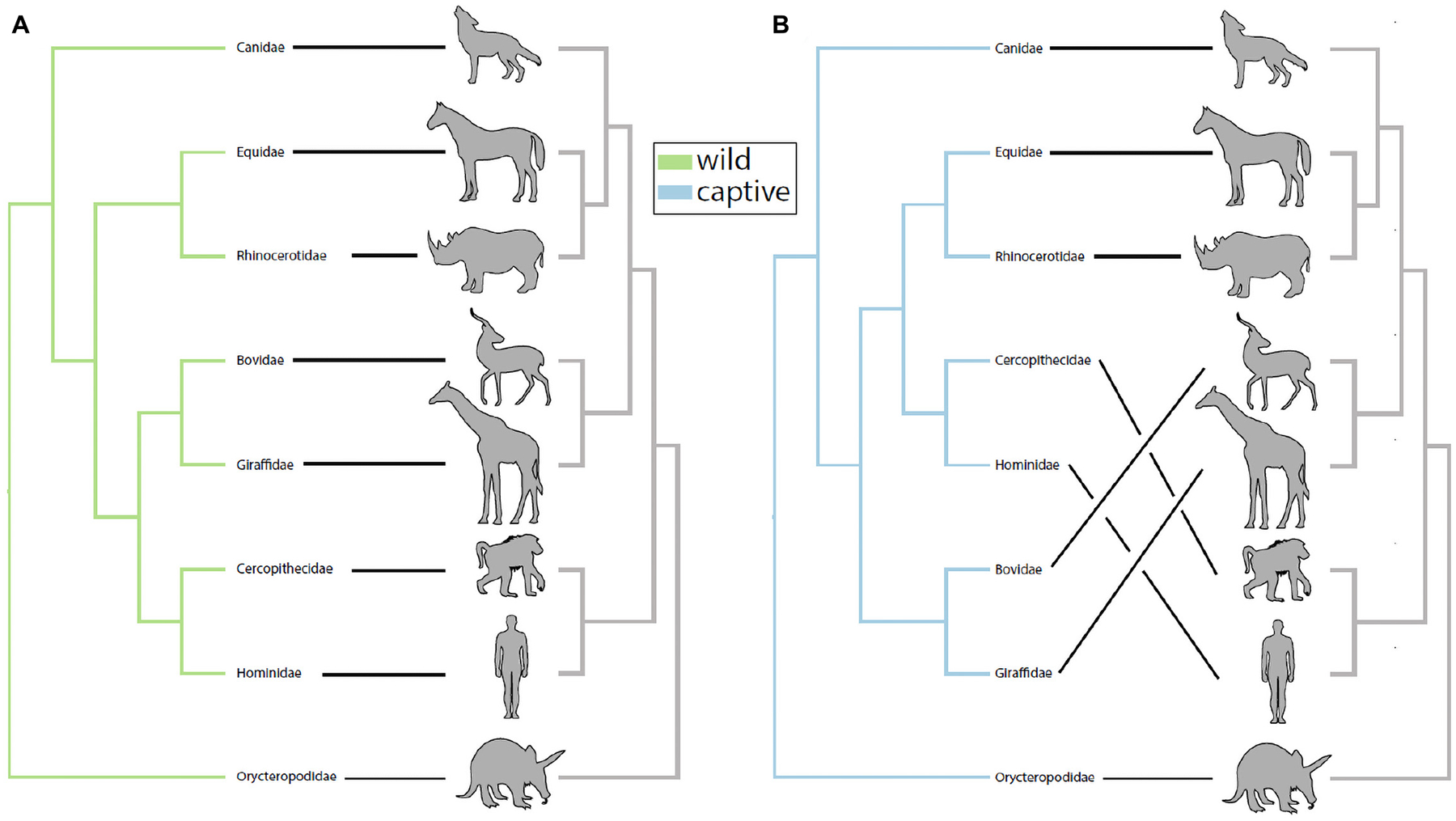
Captivity alters patterns of phylosymbiosis among mammalian families. **(A)** UPGMA-clustered microbiota dendrograms from wild mammals exhibited significant patterns of phylosymbiosis with host phylogeny (rooted Robinson-Foulds = 0.333, *P* = 0.0003). **(B)** UPGMA-clustered microbiota dendrograms from captive mammals also exhibited significant patterns of phylosymbiosis with host phylogeny (rooted Robinson-Foulds = 0.333, *P* = 0.0002). Although wild and captive trees both exhibited significant patterns of phylosymbiosis, further inspection revealed that captive Equidae and Rhinocerotidae grouped more closely with primates compared to wild confamiliars in **(A)**.

We investigated the potential impact of captivity on patterns of phylosymbiosis by comparing microbial community dendrograms and the host phylogenetic tree. Specifically, we tested whether the gut microbiota and host dendrograms exhibited more topological congruence (measure of phylosymbiosis) than expected at random, and whether wild and captive mammals differed in their patterns of phylosymbiosis. Using the UPGMA trees constructed using the Sorenson-Dice dissimilarities, we detected a statistically significant pattern of phylosymbiosis for both captive and wild trees (Figure 1). Two families–Equidae and Rhinocerotidae–clustered more closely with the primates (i.e., Hominidae and Cercopithecidae) in captivity compared to confamiliars in the wild (Figure 1). In contrast, our analysis revealed that the gut microbiota of captive Bovidae and Giraffidae retained the same phylogenetic clustering observed in wild confamiliars (Figure 1).

### Robustness and Susceptibility to Humanization of Gut Microbiota in Captive Mammals

**FIGURE 2 |:**
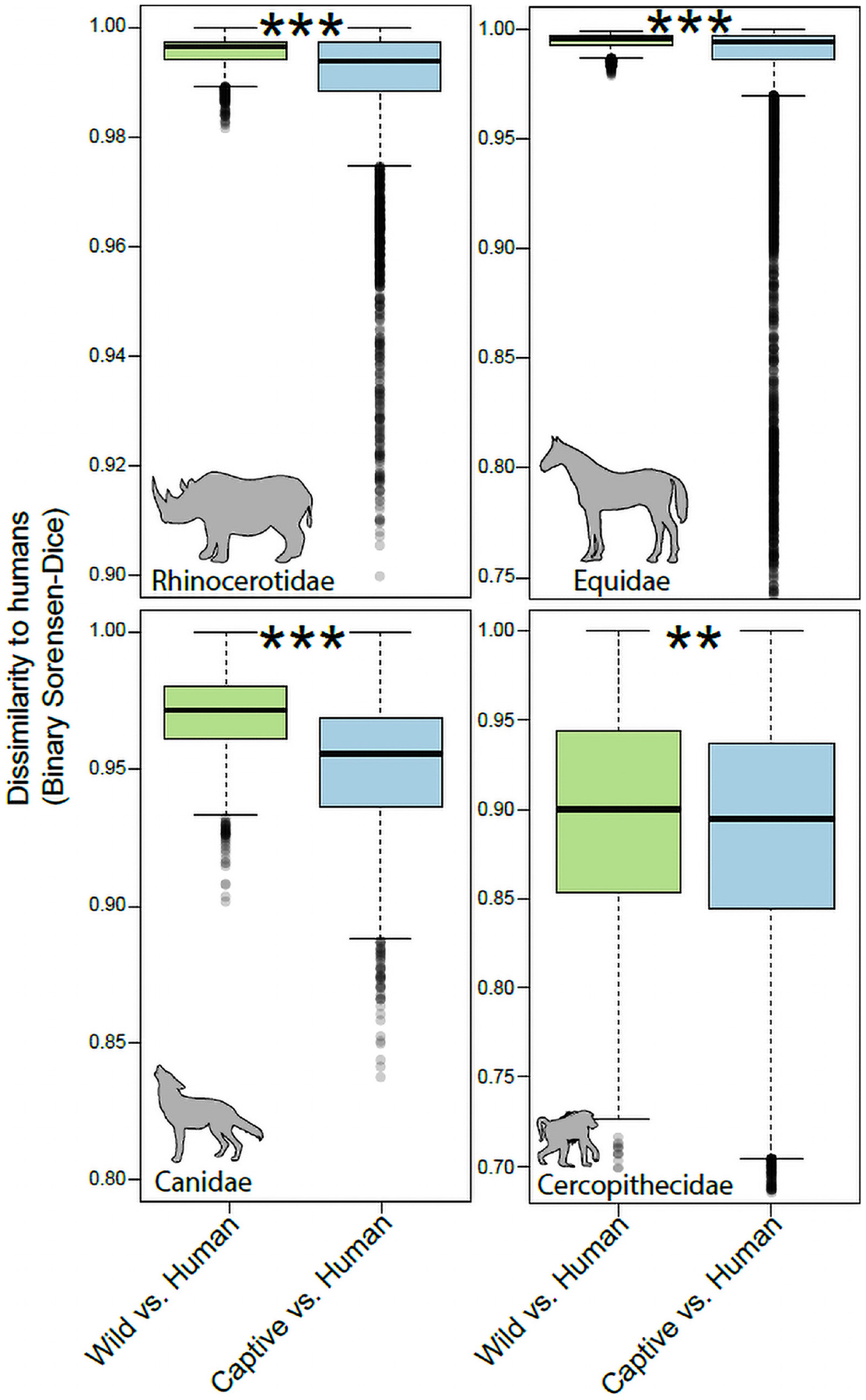
Humanization of the gut microbiota in captive Rhinocerotidae, Equidae, Canidae, and Cercopithecidae. Boxplots show microbiota dissimilarities (binary Sorensen-Dice) between humans and wild mammals (green boxes) and between humans and captive mammals (purple boxes). Each box shows median and interquartile range for a comparison including a single mammalian family. Significant differences between boxplots within each panel are denoted with asterisks; **p < 0.01, ***p = 0.001; non-parametric Monte-Carlo permutation tests.

For each mammalian family, we tested whether gut microbiota in captive individuals displayed more similar community memberships to human gut microbiota than did those of wild individuals. These tests allowed us to assess the degree of humanization of the gut microbiota in captivity for each mammalian family. Monte-Carlo non-parametric permutation tests of pairwise beta-diversity (Binary Sorensen-Dice) dissimilarities indicated that the gut microbiota of most mammalian families were more similar to the gut microbiota of humans in captivity than in the wild (Figure 2). Mammalian families that displayed significant evidence of gut microbiota humanization in captivity included the Rhinocerotidae (*p* = 0.001), Equidae (*p* = 0.001), Canidae (*p* = 0.001), and Cercopithecidae (*p* = 0.006). The sequences and taxonomic assignments of ASVs shared by captive individuals of each family and humans to the exclusion of wild individuals from the family are presented in Supplementary Table 2.

**FIGURE 3 |:**
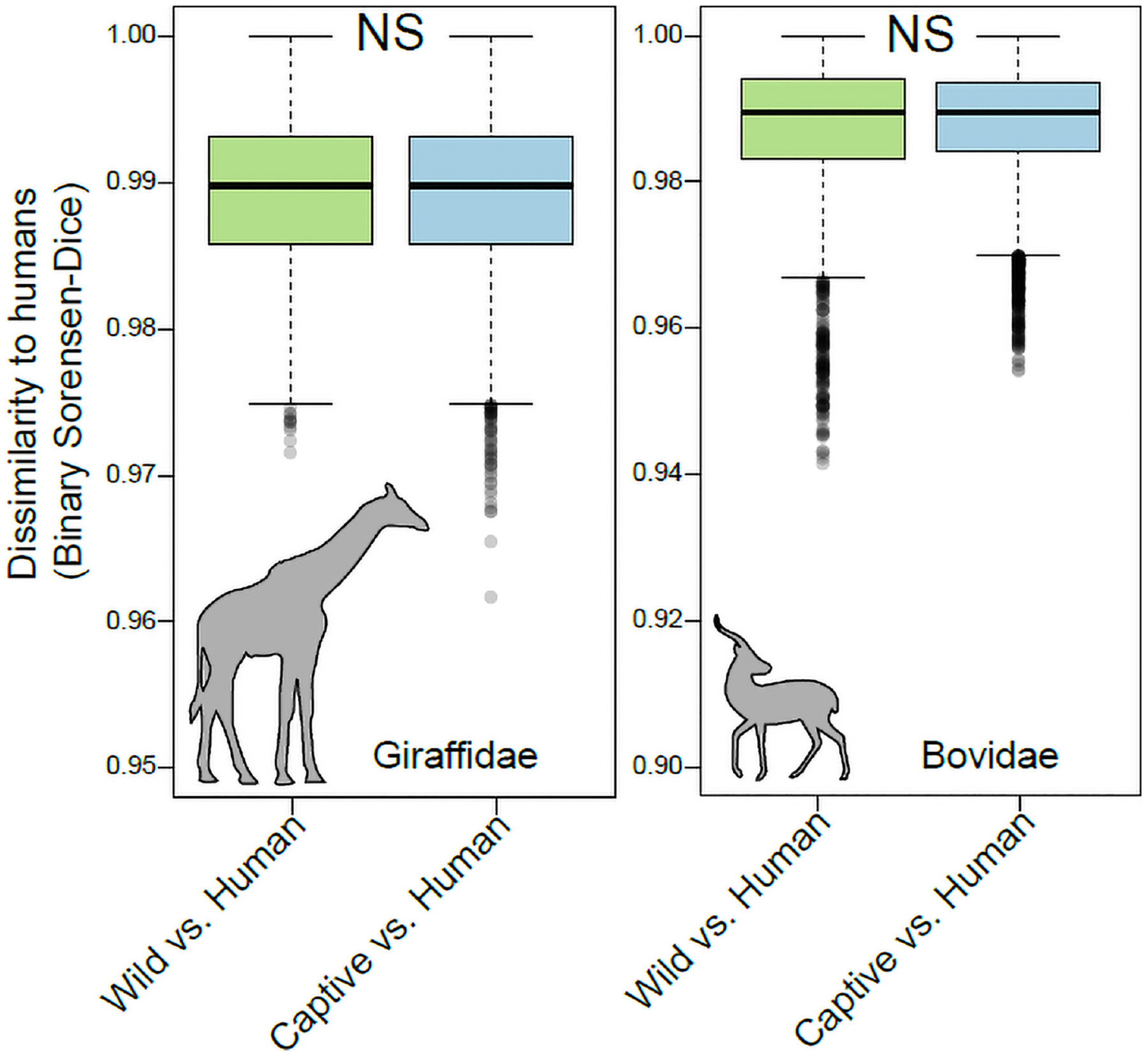
Robustness to humanization of the gut microbiota in captive Giraffidae and Bovidae. Boxplots show microbiota dissimilarities (binary Sorensen-Dice) between humans and wild mammals (green boxes) and between humans and captive mammals (purple boxes). Each box shows median and interquartile range for a comparison including a single mammalian family. Significant differences between boxplots within each panel are denoted with asterisks; NS *p >* 0.05; non-parametric Monte-Carlo permutation tests.

In contrast to the pattern observed in the Rhinocerotidae, Equidae, Canidae, and Cercopithecidae, the Bovidae and Giraffidae displayed no evidence of humanization of the gut microbiota in captivity (Figure 3). The community memberships of the microbiota in captive individuals of these families were no more similar to that of human microbiota than were those of the microbiota in wild individuals (*p* = 0.54 for Bovidae, *p* = 0.81 for Giraffidae).

### Taxonomic Distributions of Amplicon Sequence Variants Shared by Captive Mammals and Humans but Not by Wild Mammals

**FIGURE 4 |:**
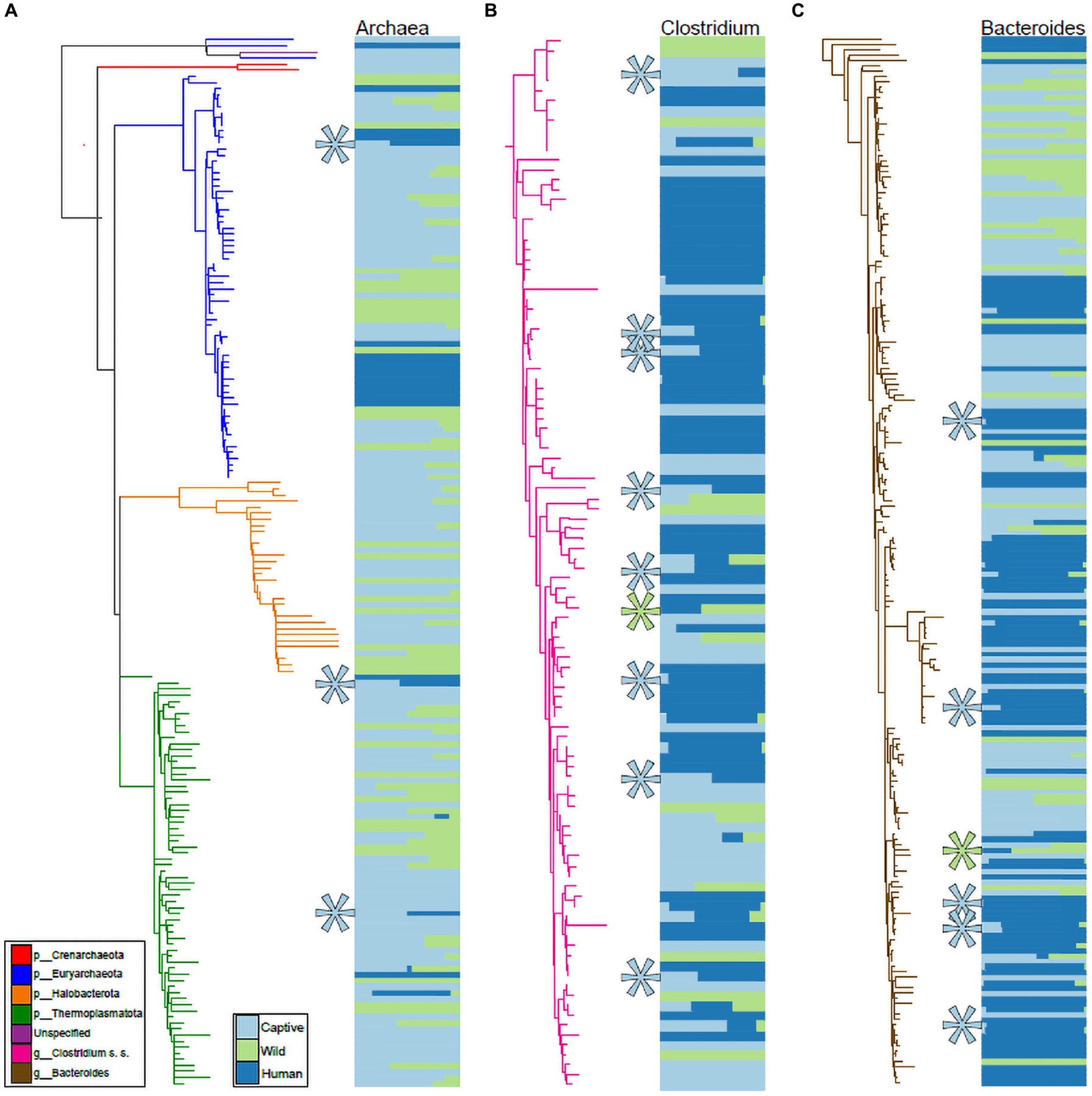
Phylogenetic distributions of Archaeal, Clostridium, and Bacteroides ASVs shared exclusively by humans and captive mammals. Phylogenies in **(A–C)** show relationships among ASVs in humans, Rhinocerotidae, Equidae, Canidae, and Cercopithecidae belonging to the Archaea **(A)**, *Clostridium sensu stricto* **(B)**, and *Bacteroides* **(C)**. Colors of branches correspond to bacterial taxa as indicated by the leftmost inset in **(A)**. Colors of horizontal bars indicate the relative abundance of each ASV in captive mammal, wild mammal, or human hosts, as indicated by the rightmost inset in **(A)**. Relative sizes of horizontal bars are scaled to reflect the mean relative abundance of the ASV in each of the three sample categories. Teal asterisks in **(A–C)** indicate ASVs shared by captive mammals and humans but not found in wild mammals (i.e., ASVs displaying distributions consistent with transfer from humans to captive mammals), whereas green asterisks indicate ASVs shared by wild mammals and humans but not found in captive mammals.

We visualized the distributions of ASVs belonging to Archaea, *Clostridium sensu stricto*, and *Bacteroides* (based on Silva 138 assignments) across humans as well as wild and captive mammals belonging to the families Rhinocerotidae, Equidae, Canidae, and Cercopithecidae. These trees and abundance plots (Figure 4) demonstrated that captive mammals exhibit a greater degree of humanization (i.e., ASVs shared by humans and captive mammals but not by wild mammals) than the opposite pattern (i.e., shared by humans and wild mammals but not by captive mammals). For example, seven ASVs belonging to *Clostridium sensu stricto* were shared by humans and captive mammals to the exclusion of wild mammals, but only a single ASV was shared by humans and wild mammals to the exclusion of captive mammals (Figure 4B).

## DISCUSSION

We found surprising variation across mammalian families in the robustness of the gut microbiota to humanization in captivity. Most of the mammalian families examined here harbored gut microbiota that were more similar to human gut microbiota in captivity than in the wild. These results are consistent with previous results from non-human primates, which display evidence of gut microbiota humanization in captivity (Clayton et al., 2016), and extend these observations to a broader diversity of host lineages spanning the mammalian phylogeny. However, in contrast to previous results from primates and the majority of mammalian families studied here, the gut microbiota of two mammalian families—Bovidae and Giraffidae—displayed robustness to humanization in captivity. This result indicates that certain mammalian taxa, in this case herbivorous Artiodactyla, did not exhibit evidence of acquisition of human-associated gut microbiota when living in captivity compared to wild confamiliars, raising questions about the mechanisms underlying this robustness and the potential implications for captive hosts.

In the Rhinocerotidae, Equidae, Canidae, and Cercopithecidae, both bacteria and archaea displayed evidence of humanization in captivity, and these patterns were driven by several bacterial taxa within these domains (Figure 4). *Clostridium sensu stricto* displayed a particularly pronounced signal of ASV sharing between humans and captive mammals to the exclusion of wild mammals (Figure 4B). This taxon contains a diversity of opportunistic pathogens, including the leading cause of mortality from bacterial gastrointestinal infections in humans *C. difficile*. Previous work has found that *C. difficile* infections can also cause mortality in captive mammals (Rolland et al., 1997). Our results suggest the possibility that these infections in captivity may stem from human sources. However, it is also possible that lifestyle factors in captivity promote the proliferation of these *C. difficile* lineages. Further metagenomic sequencing and strain tracking will be required to differentiate among these hypotheses. Moreover, this observation motivates further research into fecal transplants from wild conspecifics into captive mammals as a strategy for mitigating the effects of captivity on the gut microbiota, as fecal microbiota transplant experiments have been shown to be effective treatments for *Clostridium* infections in humans (Hvas et al., 2019).

Humanization of the gut microbiota in captivity may have consequences for animal health that subvert the aims of wildlife rehabilitation and conservation programs. For example, the routine administration of antibiotics in captivity can disrupt native microbiota (Dahlhausen et al., 2018) and therefore could allow for human-associated microbiota to opportunistically colonize the host. Intraspecific co-habitation can homogenize microbiomes between species (e.g., Song et al., 2013; Lemieux-Labonté et al., 2016), which could potentially exacerbate the mismatch between hosts and their native microbiota. Conversely, previous work has shown that facilitating conspecific interactions increases microbial transmission between individuals and improve microbial community diversity (Nelson et al., 2013), and thus could help to maintain natural microbiota and ameliorate the negative effects of captivity. Further, recent work has demonstrated that captive housing that incorporates native microbial reservoirs and diets can mitigate the loss of species-specific microbiota (Kohl and Dearing, 2014; Loudon et al., 2014). However, it is important to note that there are many host-specific factors that influence the microbiome, and thus characterization and screening of microbial reservoirs must be conducted to ensure that introduction of these materials will not result in off-target effects on native microbial communities or adverse health outcomes.

In addition to the potential adverse consequences for animal health, the observation that certain microbial lineages appear to colonize both humans and animals raises the possibility for gut microbiota transmission from captive animals into humans, a processes that could lead to zoonotic infections in humans. However, this possibility of gut microbiota transmission from captive mammals into humans has not been tested. Future work that samples humans and captive mammals at multiple facilities will have the opportunity to test whether human microbiota converge with captive mammals within these facilities.

The observation that populations of Bovidae and Giraffidae studied here do not display evidence of gut microbiota humanization indicates that certain host taxa are more robust to this effect of captivity than others. One possibility underlying this result is that the herbivorous diets of Bovidae and Giraffidae may differ substantially from those of humans, whereas omnivorous mammalian families may be less susceptible to colonization by human-derived bacteria. However, previous work in primates has shown that, at least for the genera *Alouatta*, *Colobus*, *Cercopithecus*, *Gorilla*, and *Pan*, folivore gut microbiota experience larger compositional shifts in captivity than do non-folivore gut microbiota (Frankel et al., 2019). This previous result appears to contradict the result reported here that folivorous Bovidae and Giraffidae displayed relative robustness to gut microbiota humanization in captivity, possibly due to major differences in digestive physiology between primates and artiodactyls. Resolving the association between host diet and the propensity for gut microbiota humanization in captivity will require further studies that sample a broader range of host taxa and assess this relationship in a comparative phylogenetic framework.

## CONCLUSION

Here, we leveraged hundreds of publicly available gut microbial inventories to demonstrate that captivity humanizes the mammalian gut microbiome and that the degree of humanization differs across the mammalian phylogeny. Specifically, we showed that herbivorous artiodactyls are remarkably robust to humanization compared to other mammalian families. These findings indicate that the mechanisms influencing susceptibility to humanization in captivity vary across the mammalian phylogeny. While more work is needed to elucidate the mechanistic basis of this phenomenon, our study advances understanding of microbiome humanization with implications for future experimental work and wildlife management programs.

## Supplementary Material

SM

## Data Availability

The datasets presented in this study can be found in online repositories. The names of the repository/repositories and accession number(s) can be found in the article/ Supplementary Material.
