# Supplementary material for: Robustness of Mammalian Gut Microbiota to Humanization in Captivity
Source: Front Ecol Evol. Author manuscript; Available in PMC 2022 Mar 14. (PMC8920477; doi:10.3389/fevo.2021.785089)
Supplement: SM [file NIHMS1786561-supplement-SM.zip › Data_Sheet_1_Robustness of Mammalian Gut Microbiota to Humanization in Captivity.DOCX]

Supplementary Materials

**Figure S1.** **Robustness to humanization of the gut microbiota in captive Orycteropodidae.** Boxplots show microbiota dissimilarities (binary Sorensen-Dice) between humans and wild Orycteropodidae (red boxes) and between humans and captive Orycteropodidae (blue boxes). Each box shows median and interquartile range. Significant differences between boxplots within each panel are denoted with asterisks; *** *p* = 0.001; nonparametric Monte-Carlo permutation tests.

**Table S1. Metadata for samples from humans and wild and captive mammals.**

**Table S2. Combined ASV table of human and mammalian gut microbiota**

**Table S3. Taxonomic assignments of ASVs based on the Silva 138 database.**
